# Complete Genome Sequences of Cluster A Mycobacteriophages BobSwaget, Fred313, KADY, Lokk, MyraDee, Stagni, and StepMih

**DOI:** 10.1128/genomeA.01182-17

**Published:** 2017-10-26

**Authors:** Kristen A. Butela, Susan M. R. Gurney, Heather L. Hendrickson, Janine M. LeBlanc-Straceski, Anastasia M. Zimmerman, Stephanie B. Conant, Nikki E. Freed, Olin K. Silander, Joshua J. Thomson, Charlotte A. Berkes, Cristina Bertolez, Courtney G. Davies, Amber Elinsky, Alison J. Hanlon, Juliette Nersesyan, Payal Patel, John Sherwood, Tiffany Tieu Ngo, Kathryn A. Wisniewski, Kathrine Yacoo, Paul M. Arendse, Nathan W. Bowlen, Jasmina Cunmulaj, Julie L. Downs, Charlee A. Ferrenberg, Alexandra E. Gassman, Cody E. R. Gilligan, Emily Gorkiewicz, Christopher Harness, Anthony Huffman, Christina Jones, Anna Julien, Alexis E. Kupic, Sayonara F. Latu, Thomas J. Manning, Danielle Maxwell, Merrimack College SEA-PHAGES Annotators 2016, Catherine E. Meyer, Madeleine Reardon, Matthew Slaughter, Royce Swasey, Rebecca I. Tennent, Victoria Torres, Tamia Waller, Rachel M. Worcester, Brooke L. Yost, Steven G. Cresawn, Rebecca A. Garlena, Deborah Jacobs-Sera, Welkin H. Pope, Daniel A. Russell, Graham F. Hatfull, Jacob D. Kagey

**Affiliations:** a Division of Natural and Health Sciences, Seton Hill University, Greensburg, Pennsylvania, USA; b Department of Biology, Drexel University, Philadelphia, Pennsylvania, USA; c Institute of Natural and Mathematical Sciences, Massey Phage Whānau, Massey University, Auckland, New Zealand; d Department of Biology, Merrimack College, North Andover, Massachusetts, USA; e Department of Biology, College of Charleston, Charleston, South Carolina, USA; f Biology Department, College of Engineering and Science, University of Detroit Mercy, Detroit, Michigan, USA; g Division of Integrated Biomedical Sciences, University of Detroit Mercy School of Dentistry, Detroit, Michigan, USA; h Department of Biological Sciences, University of Pittsburgh, Pittsburgh, Pennsylvania, USA; i Biology Department, James Madison University, Harrisonburg, Virginia, USA

## Abstract

Seven mycobacteriophages from distinct geographical locations were isolated, using *Mycobacterium smegmatis* mc^2^155 as the host, and then purified and sequenced. All of the genomes are related to cluster A mycobacteriophages, BobSwaget and Lokk in subcluster A2; Fred313, KADY, Stagni, and StepMih in subcluster A3; and MyraDee in subcluster A18, the first phage to be assigned to that subcluster.

## GENOME ANNOUNCEMENT

*Mycobacterium smegmatis* mc^2^155 is a well-characterized actinobacterium that is used as a host for bacteriophage discovery (1). There are currently over 1,300 completely sequenced mycobacteriophages that have been discovered by participants in the Howard Hughes Medical Institute Science Education Alliance-Phage Hunters Advancing Genomics and Evolutionary Science (SEA-PHAGES) program (2, 3). These phages are considerably diverse, forming 24 clusters (clusters A to Z) and six singletons (those without close relatives). Cluster A is the largest cluster, with over 500 individual phage members, and it is subdivided into multiple subclusters based on overall sequence relationships (4, 5).

Seven mycobacteriophages were isolated from soil or compost samples using either enrichment culture or direct plating with the bacterial host *M. smegmatis* mc^2^155 at 26 to 37°C. All seven phages are morphologically members of the family *Siphoviridae*. The genomes were sequenced using the Illumina MiSeq platform with 150-bp reads and assembled using Newbler and Consed, with at least 300-fold coverage (6, 7). The genomes were annotated using DNA Master (http://cobamide2.bio.pitt.edu), Glimmer (8), GeneMark (9), Starterator, Phamerator (10), HHPRED (11), BLASTp searches against the NCBI nonredundant and actinobacteriophage (http://phagesdb.org) databases (12, 13), Aragorn (14), tRNAscanSE (15), and PECAAN (http://pecaan.kbrinsgd.org). Phage features are listed in Table 1.

**TABLE 1:** Seven newly isolated cluster A mycobacteriophages

Phage name	Accession no.	Length (bp)	G+C content (%)	No. of ORFs^a^	No. of tRNAs	Cluster	Location of isolation
BobSwaget	MF185727	50,400	63.3	89	1	A2	Andover, MA, USA
Lokk	MF324899	51,008	63.4	88	1	A2	Andover, MA, USA
Fred313	MF373840	50,053	64.0	85	2	A3	Chesterfield, MI, USA
KADY	MF185729	50,898	64.2	90	3	A3	Warren, MI, USA
StepMih	MF185733	50,841	64.0	93	3	A3	Auckland, New Zealand
Stagni	MF185732	50,856	64.0	89	3	A3	Livonia, MI, USA
MyraDee	MF141539	50,514	62.7	94	0	A18	Saltsburg, PA, USA

a ORFs, open reading frames.

All of the genomes are approximately 50 kb long and have nucleotide sequence similarities to those of cluster A phages. BobSwaget and Lokk are grouped into subcluster A2, and Fred313, KADY, Stagni, and StephMih are grouped into subcluster A3, according to their nucleotide sequence similarities. MyraDee is not closely related to phages in any particular subcluster and thus is the founding member of subcluster A18. We note that although Stagni and StepMih were isolated from geographically distinct locations, they share 99.8% nucleotide identity over their genome length. All of the phages have a typical cluster A genome organization, with the virion structure and assembly genes in their left arm and regulatory and replication functions in their right arm (16). However, they differ in the number and types of tRNA genes near their left genome end; Kady, Stagni, and StepMih have three tRNA genes (tRNA^Asn^, tRNA^Leu^, and tRNA^Trp^), Fred313 has two (tRNA^Asn^, tRNA^Trp^), BobSwaget and Lokk have one (tRNA^Gln^), and MyraDee has none.

All seven phages have features consistent with temperate lifestyles and encode putative immunity repressors related to L5 gp71 (17). However, they differ in the genes near the centers of the genomes that confer prophage maintenance. For example, Fred313, Stagni, and StephMih code for integrases of the tyrosine recombinase family, whereas KADY and MyraDee code for serine integrases (18). In contrast, BobSwaget and Lokk have *parABS* partitioning systems, as described for several other cluster A phages (19, 20), although their ParA and ParB proteins share only 62% and 48% amino acid sequence identities, respectively. Lokk, but not BobSwaget, codes for a putative RepA protein (gp36) that is implicated in extrachromosomal prophage replication and that shares 71% amino acid identity with phage CRB1 RepA (20). MyraDee codes for a putative ArdA-like antirestriction protein (gp87).

### Accession number(s).

The genome sequences reported here have been deposited in GenBank under the accession numbers given in Table 1. The versions described here for these phages are the first versions reported.
